# Success in the workplace: From the voice of (dis)abled to the voice of enabled

**DOI:** 10.4102/ajod.v3i1.99

**Published:** 2014-11-20

**Authors:** Gloria Marsay

**Affiliations:** 1 Independent psychologist in private practice

## Abstract

The intention of this article is twofold; first to encourage a shift in seeing ‘the disabled’ not as people with disabilities but rather as people with unique abilities. Secondly, to explore ways of facilitating gainful employment for these uniquely abled people. The term disability is examined against a backdrop of definitions including the definition postulated by the International Classification of Functioning. In this article, the life experiences of a purposive sample of people with (dis)abilities who have been successful in the world of work are explored. A narrative approach gives voice to their experiences. Quotes from the participants’ responses are used to illustrate the common themes that emerged relating to their experiences. These themes are resonated against a backdrop of relevant literature. If disabled people are enabled to recognize and use their unique abilities, as well as develop various self-determination skills, imagine the endless possibilities which could arise for them and society in general.

## Introduction

The United Nations Convention on the Rights of Persons with Disabilities (CRPD) (United Nations 2008) facilitated the implementation of programmes and policies regarding the rights of people with disabilities in South Africa. However, it appears that there continues to be significant gaps in knowledge regarding the situation of people with disabilities, their families and their environment. Thus, opportunities for success are limited (Department of Women, Children and People with Disabilities [DWCPD] 2013; Department of Social Development [DSD], DWCPD & UNICEF 2012; Eide & Ingstad 2013; Wehmeyer 2013a).

The intention of this article is twofold: firstly, to encourage a shift in attitude towards people with disabilities, regarding them not as disabled, but rather as people with unique abilities. Secondly, to reveal and discuss some of the common themes that people with disabilities have used to describe their experiences in the world of work.

The International Classification of Functioning, Disability and Health (ICF) classification system uses, to the extent possible, neutral language to name its components and categories. The use of neutral language is helpful and also challenging. For the purpose of this article, I choose to engage in positive and preferred language, and to refer to people with (dis)abilities because the focus is on individual ability.

### Historical views of disability

The view of people with (dis)abilities has shifted during the last 60 years. The catalyst for this shift could be attributed to the droves of veterans who returned to their home countries in Europe with disabilities after serving in World War II. Until the 1950s, people with (dis)abilities were considered to be dysfunctional and were often institutionalised. Since then, two major models have been debated, namely the Medical and Social Models. The Medical Model is best summarised by referring to the International Classification of Impairments, Disabilities and Handicaps (ICIDH) developed by the World Health Organization (WHO 1980). This model focusses on disability as an individual pathological problem, and promotes the view of a disabled person as dependent and in need of care and cure. Hence, people were systematically excluded from society. Eventually, people with disabilities began to challenge the way they were treated, giving rise to the Social Model, which draws attention to physical, social and environmental barriers that construct disability. The Disabled People’s Movement argues that the ‘cure’ to the problem of disability lies in the restructuring of society to reduce barriers that prevent people with disabilities from realising their potential. In response to these models, the World Health Organization developed the International Classification of Functioning, Disability and Health (ICF) which provides a multidimensional framework that is widely used today (WHO 2001). The change from focussing on the individual disability as pathological, to focussing on the individual within the context of the environment and social community has important implications for developing policies pertaining to (dis)ability and has been richly debated (Buntinx 2013; Brueggemann 2013; Eide & Ingstad 2013; Oliver 2009; Shogren 2013; Watermeyer *et al*. 2006; Wehmeyer 2013b; Wilson & Lewiwcki-Wilson 2001). Recently, (dis)ability has been examined through the lens of strengths-based Positive Psychology. Wehmeyer (2013b) states the following:

The historical view of disability as pathological has run its course, although it remains far too prevalent. The success of people with disabilities in all aspects of life, aided by civil protections and equal opportunities, has made pathology-based understandings of disabilities irrelevant or inaccurate. It is well past time to begin to consider disabilities using a strengths-based focus. (p. 5)

The strengths-based approach of Positive Psychology provides a platform for discussion in this article.

### Defining disability

Defining disability is controversial and difficult. The ICF views disability as a complex phenomenon and provides a multidimensional framework incorporating medical and rehabilitative interventions with environmental and social interventions in a more optimistic way. The advantage of this framework is that it incorporates all aspects of a person’s life, including medical (body function and structure), social (ability to participate), environmental factors (the person within the context of his or her physical world) and personal factors (race, gender, age and education). Although the ICF-model attempts to be culturally neutral, the question is whether such neutrality is possible. Different kinds of impairments are understood differently, and have different consequences in different cultures (Brueggemann 2013; DWCPD 2013; Eide & Ingstad 2013; Watermeyer *et al*. 2006).

According to Bach (2013), 80% of people with (dis)abilities live in developing countries. Seeing that data collection is difficult, not much research has been possible. Eide and Ingstad (2013) report that there are substantial gaps in services for people with disabilities, and that disability is associated with a lower level of living. The fact remains there is a link between poverty and disability and that disability affects millions of families in developing countries (DSD *et al*. 2012; Eide & Ingstad 2011; Eide & Ingstad 2013; Filmer 2008; Priestley 2006; Watermeyer *et al*. 2006; WHO 2011). The construct of (dis)ability can be interpreted as a form of social inequality resulting from oppressive social structures rather than from individual difference or biology (Balcaza *et al*. 2009; Brueggemann, 2013; Eide & Ingstad, 2013; Priestley 2006).

### The South African context

South Africa is described as a rainbow nation, which means that it is a home to a plethora of different cultures that influence the interpretation of (dis)ability. The perception of ‘disability’ is exacerbated in the South African context by historical and political structures. The history of South Africa is woven with tales of people in authority, dictating to others what they need, and what is good for them (Hansen & Sait 2011). The policy of segregation from the past, and the present *Employment Equity Act* have given rise to two approaches to (dis)ability in the South African context: the broad definition (disability as discrimination) and the narrow definition (disability as impairment) (Hansen & Sait 2011; Van Deventer 2011). These two approaches cloud perception and interfere with proper reporting, thus blurring quality data on disability in the South African context. Some people are unable to recognise and acknowledge that they have a disability. Others, fear stigmatisation and consequently fail to report (dis)ability. Figures received from specific disability organisations often contradict those received from Statistics South Africa (2010; cf. DWCPD 2013); for example, the Disability Rights Commission (DRC) Report of 2007, states that: [*S*]tatistics, where available, suggest that disabled people are under-represented or are present but not disclosing their health or disability status and so are not represented in the figures. (p. 20).

This view is echoed by Van Deventer (2011) who suggests that statistics vary between 2% and 12%, and that there is disparity between all the organisations that present statistics regarding people with (dis)abilities who are employed. Despite commitments from the National Skills Development Strategy (NSDS) to increase opportunities for training and skills development for people with (dis)abilities South Africa is still far from achieving its goal in this regard (Department of Higher Education and Training [DHET] 2010; Soudien & Baxen 2006). Despite the set target to employ a minimum of 3% of people with (dis)abilities, the figure for employed people with (dis)abilities dropped from almost 1% in 2009 to 0.5% in 2011 (Van Deventer 2011).

South Africa adopted a policy of inclusion in education and integrated learners with special needs into ‘mainstream schooling’. This move has not always benefitted learners with special needs because of the diversity of difficulties, exacerbated by the fact that not all educators are trained to recognise and deal with (dis)abilities (Dalton, Mckenzie & Kahonde 2012). Data from 22 of the 23 public universities shows that 5807 students with disabilities were enrolled in higher education institutions in 2011, accounting for only 1% of total enrolment (DHET 2014). This decrease could be attributed to the limited opportunties for education and training. The World Health Organization Report (2011) states that:

In South Africa it is thought that school attendance and completion are influenced by the belief of school administrators that disabled students do not have a future in higher education. (p. 216)

However, it would appear that cognisance has been taken of this disturbing fact. Blade Nzimande, Minister of Higher Education and Training, speaking at the launch of a white paper on post-school education and training in January 2014, stated that:

Despite attempts to integrate disability into the broader policy arena, currently there is no national policy on disability to guide education and training institutions in post-school domain. The management of disability in post-school education remains fragmented and separate to that of existing transformation and diversity programmes at the institutional level. Individual institutions determine unique ways in which to address disability, and resourcing is allocated within each institution according to their programme. Levels of commitment toward people with disability vary considerably between institutions, as do the resources allocated to addressing disability issues. TVET (Technical Vocational Education and Training) colleges in particular lack the capacity, or even the policies, to cater for students and staff with disabilities. (DHET 2014:8)

This statement highlights the difficulties experienced within education and training facilities in South Africa. Furthermore, Nzimande stated that the Department of Higher Education and Training’s disability funding was underutilised in 2010 and 2011 at levels of only 47% and 55% of available funding respectively (DHET 2014:8).

## Motivation

There have been several calls for research to promote the rights and participation of disabled people in our society (AfriNEAD 2009; DHET 2014; DWCPD 2013; Eide & Ingstad 2013; WHO 2011). Research is urgently needed to move disability up the economic development agenda (World Bank 2000). The slogan Disabled People South Africa (DPSA) adopted is ‘Nothing about us, without us’. Participation of people with (dis)abilities is regarded as an important aspect of the new paradigm on (dis)ability, and has important implications for the way in which research is done. One cannot overemphasise the need for collaboration between people who have (dis)abilities, professionals who assist people with (dis)abilities, civil society and state institutions. More knowledge and a new understanding are gained by engaging in conversation with people who have (dis)abilities. Oliver (2009) states that:

If disabled people left it to others to write about disability, we would inevitably end up with inaccurate and distorted accounts of our experiences and inappropriate service provisions and professional practices based upon these inaccuracies and distortions. (p. 9)

There is a danger that research may become oppressive. Therefore, the aim of this study is to engage in what is referred to as ‘emancipatory research’ using indigenous knowledge (Barnes & Mercer 1997; Barnes, Oliver & Barton 2002; Moore, Beazley & Maelzer 1998; Oliver 2009). The reality is that people with (dis)abilities are their own best advocates (Barnes & Mercer 1997; Barnes *et al*. 2002; Eide & Ingstad 2013; Filmer 2008; Oliver 2009; Watermeyer *et al*. 2006).

I work as a psychologist assisting people with (dis)abilities to position themselves in the world of work. In response to a need to improve my way of working, and in an attempt to stimulate further conversation around effective policies and practicies, I have undertaken this study. I could be classified as (dis)abled. However, I have offered resistance to this theoretical classification and have endeavoured to overcome difficulties that prevent me from doing the work I wish to do. In fact, perhaps the need to overcome these difficulties may inspire and assist the work I do.

## Research methodology

This is a qualitative descriptive study using a narrative approach. This approach was chosen because it allows the experiences of people with (dis)abilities in the world of work to be described, thereby giving greater meaning to practice. Narrative inquiry has a distinguished history and is increasingly used in studies that describe social experience (Clandinin & Connelly 2000; Josselson & Lieblich 1995; Lieblich, Mashliach & Zibler 1998; Riessman 2008; Sandelowski 2000).

Narrative inquiry successfully captures personal and human dimensions that cannot be quantified into dry facts and numerical data (Clandinin & Connelly 2000). As a researcher, I seek credibility based on accountability, trustworthiness and dependability. A process of reflexivity was used, which makes the researcher aware of her own experiences, perceptions and interpretations and how these may influence the way she hears what the participants are telling. Ethical considerations governing this study include the following:

- Participation in this project was entirely voluntary.
- There was neither cost nor benefit for the participants.
- The participants were entitled to read the draft of this paper and make comments.
- Confidentiality was and is respected at all times.

### Describing the participants

Oliver (2009:5) states that ‘the link between personal experience and what people write cannot be ignored and should not be denied’. A purposive sample was selected (*N* = 25 with 14 men and 11 women from diverse cultures). All the participants have received education and training in marketable skills. They are all gainfully employed and live in urban areas. The average age of the participants is 37.4 years; however, two participants did not reveal their age.

Various categories of (dis)ability are represented, and often more than one (dis)ability is stated for each person. It is important to note that the primary (dis)ability is used to compile the frequencies. Eleven participants were born with the condition and 14 participants acquired their condition through illness, injury or traumatic life events:

- physical difficulties (*N* = 9) – four of these people have difficulty with mobility
- emotional difficulties (*N* = 5) – specifically depression and anxiety
- learning difficulties (*N* = 6) – three with Attention Deficit Hyperactive Disorder (ADHD), two with Attention Deficit Disorder (ADD), and one person falling within the autism spectrum.
- sensory impairment (*N* = 3) – two people with visual impairment and one with hearing impairment
- chronic illness (*N* = 2) – one person with epilepsy and one with rheumotoid arthritis.

#### Data collection

A biographical questionnaire was completed. Then, prompted by moderately structured open-ended questions, participants were invited to describe their experiences in the world of work. Some participants preferred to tell their experiences orally, others preferred to write down their responses.

#### Making meaning of the data

Qualitative thematic content analysis was used to identify the themes that emerged from the responses. Sometimes the themes overlapped and hence were modified in the course of analysis as it became necessary to accommodate new data and new insights (Lieblich *et al*. 1998; Riessman 2008; Sandelowski 2000). The themes used to describe the experiences of the participants were named, confirmed by counting and then summarised using descriptive statistics.

Located in a hermeneutic circle of re-interpretation, narratives with common story elements can be reasonably expected to change from telling to telling, making the idea of empirically validating them for consistency or stability completely alien to the concept of narrative truth (Clandinin & Connelly 2000; Josselson & Lieblich 1995; Lieblich *et al*. 1998; Riessman 2008; Sandelowski 2000).

Whilst acknowledging that no description is free of interpretation, it is also important to remember that although a particular theme was not mentioned, it does not mean that it was not experienced by the person. Therefore, it is difficult to offer absolute frequencies. Themes that emerged from each question are summarised below, illustrated with some direct quotations. A discussion reflecting these responses against the background of literature follows thereafter. The reader is invited to reflect his or her experiences against the experiences of the participants.

## Descriptive summary of data

### First question: How did you become employed or self-employed? Please describe the process

More than one way of entering the world of work was mentioned by all the participants and frequencies are difficult to determine. There is nothing unusual about the approaches mentioned, however there were some interesting responses which are illustrated. The following themes emerged:

- placement through agencies that specialise in placing people with (dis)abilities
- finding employment through social networking
- joining the family business
- becoming self-employed.

#### Placement through agency

It was interesting to note that some of the participants experienced their interviews as hostile. It would appear that the prospective employer, and in one case the placement agency, was not sensitive to the unique needs of the participant, as illustrated by the following quotations:

L: ‘I applied for a job through a recruitment agency that got work for disable people. The job was to stand all day and greet people. Unfortunately that didn’t last long as I am not able to stand all day because of rheumatoid arthritis.’

Ca: ‘Interview was difficult. All would go well in the interview until I mentioned I am hard-of-hearing. That word alone scared them.’

#### Social Networking

L: ‘I was fortunate enough that a distant family member owned her own little electrical wholesale company and she offered me a job as a “girl Friday” and receptionist. I was very lucky as they gave me in-house training. I eventually worked my way up to being a debtors/creditors clerk. After that, it was a little easier getting jobs as I had extensive experience.’

#### Family business

R: ‘After school I joined my father in his building business In 2008 we bought a farm where we farm with cattle and we still build occasionally.’

#### Self-employed

Three participants (12%) are self-employed. Both the participants had worked for a period of time within their field of interest before moving into a position of being self-employed:

B: ‘Whilst studying at university, I was offered a part time position at a media company during the university holiday. I enjoyed the work very much – more than the studying – as it was involved in a field I always wanted to be in. I was kept on as a freelancer for several years, performing multiple tasks in various different disciplines. After a few years working as a freelancer on a permanent contract, I decided to start my own company and have been working successfully on my own for the last six years.’

### Second question: What helped you to become and remain successfully employed?

The major themes that emerged from this question reflect many of the constructs described in the paradigm of Positive Psychology (Wehmeyer 2013a). The main themes were:

- choosing or creating an enabling environment
- self-determination and good work ethic
- support structures (personal, organisational, environmental and spiritual)
- self-knowledge.

#### Enabling environment

All the participants mentioned that they either chose or created an enabling environment:

G: ‘You need to find the right environment … doing a project based on my best capabilities. The closer you get to what you are good at and what interests you, you’ll just be on fire.’

Co: ‘My lecturers at medical school accommodated my needs since they were enlightened as doctors and appreciated my coping skills and recognised my potential.’

Lv: ‘My job now is great as my bosses have been very understanding. They know I have days where I am too depressed to come in to work and they allow me work from home days. They constantly give me feedback and tell me how I am doing and praise me. This keeps me employed. I still consider quitting very often, but it is the support I have from them that keeps me coming to work knowing that I am valued.’

L: ‘Doing work that is aligned with your soul. A job that gives you satisfaction, even if for less money. It must be a job that can allow flexibility to allow for the intensely bad times.’

#### Medication and support from professionals

All the participants received professional support in one form or another as they had all been diagnosed and categorised with a condition; some received medication. For others, it was counselling that assisted them. The following quotations illustrate the specific kind of assistance received from professionals:

L:‘ I make sure I have all the medicines and creams I need with me every day in case my muscles go into spasm.’

F:‘ I have been on medication for my depression since the age of 15 which helps to keep my mood more stable and regulated than I am able to do on my own. Working in such a pressured job, I ensure that I get regular supervision within work. I have a fantastic psychiatrist who knows me and what I’ve been through very well as well as a psychologist who is always willing to listen. It took lots of counselling and antidepressants to get me to the stage where I was stable enough to be able to return to school and later on hold down a job.’

Lv: ‘Ongoing support from a psychologist and medication.’

#### Self-determination and good work ethic

Self-determination was a theme mentioned by 76% of the participants and 76% of the participants (not always the same participants) also mentioned good work ethic:

P: ‘School examination results … I also had a very high work ethic which meant that I always tried to be better at my job than my peers.’

Ca: ‘Work harder, research more, get full support from colleagues, ask for support and guidance when I needed help.’

Kh: ‘Promotion did not come easy because of my disability but it was due to dedication and working an extra mile.’

J: ‘Hard work and proving to people that being in a wheelchair would not stop me from doing my best. The right attitude.’

Lv: ‘I have struggled to stay employed. People within my various work places would be shocked to hear this – but every day has been a struggle emotionally and psychologically. Debilitating anxiety that has paralysed me to the point of not being able to breathe … pushing through and forcing myself to do normal tasks.’

#### Support structures

Sixty percent of the participants mentioned that they actively sought social support at work, which was helpful for them:

Kh: ‘One of my colleagues offered to go with me to visit schools [*part of the participant’s work*]. She reminded me that I am not a product of lazy people because she knew me before and that I was working hard. That was a breakthrough that got me to venture out to schools again.’

FM: ‘My mentor is very patient with me, sometimes I take along time to understand some of the things but she doesn’t give up on me.’

Jw: ‘The key to my employment was the risk that the headmaster was willing to take and the lack of pressure to conform to traditional teaching methods.’

Kh: ‘I had full support of the management which made me feel valuable and it was a motivator to realise that I am not different from other people. I got support from my superiors by providing me a personal driver who will assist me to carry out my duties like before. It came because I had to show them that I was enthusiastic to accomplish my mission in this world.’

#### Support from family

Forty-four percent of the participants mentioned that social support from family and friends was helpful:

F: ‘I have an amazing family and very supportive friends who have stuck by me through everything I’ve been through.’

L: ‘The support of my family played a huge role in me maintaining a job.’

Kh: ‘My family especially my mother, husband and children played a major role in supporting me.’

#### Self-knowledge

Forty-four percent of participants commented that self-knowledge in terms of their own strengths and limitations assisted them to regulate and adapt their own behaviour so that they were able to work optimally.

One participant who struggles with ADHD runs his own business, with the assistance of a chartered accountant who takes care of all the financial running of the company. Another participant, who also struggles with ADD, runs his business without assistance and admits that it is disorganised and not as productive as it could be. The comparison of these two stories highlights the need for people to understand and accept their limitations, to have the courage to seek assistance, and then to focus on their strengths and interests:

F: ‘I am very aware of my own short-comings and pay close attention to my emotions and any indications that I may be headed towards depression again.’

G: ‘You also need to be clever about what you suck at and who you can use to help you. I have always paired up with someone (a colleague I am friendly with). It’s not all about feeling helpless and in need of assistance. That person will very often require your strengths.’

Good communication skills were mentioned by 36% of the participants:

JN: ‘I think that the key is communication, willingness and determination. The companies that I have worked for so far have been very accommodating. I have been able to freely express my needs. The fact that l know that it is a two way relationship has helped a lot to address issues.’

Twenty percent of the participants commented on how spirituality has assisted them:

C: ‘I attribute my success to my Lord Jesus, my Godly, Christian parents and family, Christian teachers who saw my potential and encouraged my always.’

Kh: ‘I am grateful to God for bringing such empathetic people around me. I was able to experience fulfilment in what I was doing. It made me stronger, even to stand against some discriminatory practices.’

Only one participant commented on her access to technological assistance (JAWS speech reader) and how this technology assisted her once she had been trained to use it. Eide and Ingstad (2013) state that nearly half of those who need assistive technology devices do not have access to one. The question can be posed whether this means that technological assistance is scarce or did the participants simply not make any mention of technological assistance:

Jw: ‘The only area that I needed extra assistance with was with entering marks onto the computer (because I hadn’t yet been trained on the JAWS speech reader programme).’

#### Third question: What were the obstacles you had to overcome?

It would appear that the obstacles mentioned were influenced by the nature of the (dis)ability of the person: physical, sensory, environmental or social. Themes evident in these responses were:

- special needs are not always met
- negative perception of self
- stigma and discriminatory practices
- difficult experiences in education and training.

#### Special needs not always met

All of the participants who have mobility difficulties reported hostile environments and difficulties with access and transport. Furthermore, the participants with visual difficulties also commented on transport difficulties. The participant with a hearing impairment also expressed difficulty in her work environment:

J: ‘Accessibility was a big concern for me, even though companies say they are accessible, they really are not. You need to be in the situation to understand the obstacles.’

Ca: ‘Special needs are not always met – need to make sure get what meetings are about – find ways.’

Jw: ‘I had to learn to take public transport. To be independent I initially needed a sighted person to help me to learn the routes. Then I had to swallow my pride and learn to hold up a sign showing my destination so that the relevant buses would stop.’

Kh: ‘There were numerous challenges along the way. It was not easy at all especially with accessing bathroom and other venues as there are no ramps. My old office (before the accident) was a new environment as I could not access most of the things such filing cabinets and limited my movement because the office and bathroom were small. I sat in the office I used to know, without anybody to support me as to how to adjust. I had to figure out on my own what to do because people do not know what to do for me.’

#### Stigma and discriminatory practices

Twenty-eight percent of participants commented that the attitude of others (those with whom they work and society in general was difficult):

F: ‘I suffer from depression which often is not classed as a disability as such, but rather as a mental health issue. This increases the stigma surrounding the disease and makes me and others like me less likely to talk about it and ask for help.’

Kh: ‘I was understood as requiring preferential treatment. I was harassed into taking action by being granted what is called special leave (polite way of dismissal) because the organisation could no longer bear with my presence in the office. Thank God because I have forgiven those people. One of the qualities one develops due to disability is patience and forgiveness.’

One participant commented about unfair Black Economic Empowerment (BEE) laws which made it difficult for him to obtain employment:

J: ‘Unfortunately in SA, the BEE law just is not fair, especially to a white male.’

#### Negative self-perception

Sixty-four percent of the participants described their negative self-perception as an obstacle:

H: ‘I was afraid I wouldn’t be good enough and that people wouldn’t respect me. I had to put aside my fear of failure. My mood constantly affects those around me. It is not fair for me to expect others to support and help me “pick myself up”. It is a consistent on-going struggle to remain positiveand it is hard work.’

Kh: ‘One has a fear of the unknown and what will people accept me and this paralyses you further. It is pretty tough to compete with abled people in the workplace because you constantly want to prove that disability is not an obstacle to deliver quality service.’

#### Difficult experiences in education and training

Two people with ADD (16%) commented about their learning experiences being difficult. Their teachers had not understood their specific needs and they had not reached their full academic potential. They were only diagnosed with the difficulty in adulthood. They had subsequently received treatment, which had been of benefit to them. These examples illustrate that education structures are not always supportive and enabling:

M: ‘I have only recently found out that I have ADD which explains a lot why business has not run as successfully as it should. The distraction of the ADD in my head has caused me to be disorganised and also why I didn’t get a better qualification. I wish that I had known about this condition long ago.’

B: ‘Suffering from ADHD could be a debilitating problem in an unstimulating environment. As such, formal education could be regarded as problematic, as there is very little in the way of multiple thought paths.’

### Fourth question: Please feel free to add anything more you may feel is of interest with regard to you becoming gainfully employed despite the fact that you are differently abled

- lack of awareness and the need to educate others about (dis)ability
- defying the disablist attitude
- (dis)ability has proved to be a strength.

#### Lack of awareness and the need to educate others about (dis)ability

Fifty-two percent of participants commented that they needed to educate those with whom they worked and others in their social network about their special needs and how they can best be helped:

Jw: ‘Educating others about my disability, for example: “When you greet me, please tell me who you are”; “follow my nose and not my eyes”; “may I sit in the chair where my back is to the light?”; “please walk ahead of me”. Sometimes reminding people that I am visually impaired and not brain dead in as tactful and kind a way as I can.’

J: ‘Most of all, teach people that do not have disabilities that I am not different – I just don’t walk.’

#### Defying the disablist attitude

Forty-eight percent of the participants commented on how their own change in attitude assisted them. They described how channelling their (dis)ability into a strength, and offering resistance to their own disabilist attitude, enabled them towards success:

Kh: [*After the acciden*t] ‘My doctor declared me as incapacitated and unable to go back to work again as he alleged that I was 100% disabled. I refused to believe him and I did not submit the “medically unfit” certificate to my employer.’

P: ‘My philosophy was that I never saw myself as disabled and this resulted in the people I worked with very quickly becoming ‘blind’ to my disability.’

Ca: ‘Being hard of hearing wasn’t going to prevent me from becoming the person I wanted to be, a teacher.’

#### (Dis)ability as a strength

Twelve percent of the participants consider their disability to be a strength:

B: ‘In my field, and specifically running my own business, one needs to be able to concentrate on a variety of things simultaneously, and my “disability” actually serves me well in terms of when one task is getting boring or running smoothly, my mind moves over to another, and I can jump from one task to another without interrupting a train of thought. In my industry, we’re permanently moving, changing environments and facing different problems with each new setup. There is nothing mundane about it, and there is no routine. The lack of routine could adversely affect people who require stability in order to function, but it’s the very thing that makes it easier for me to tackle, as there is always something new to stimulate the mind.’

G: ‘The energy – be it physical or mental that you once were made to believe was a disability will help you to think on your feet, be seen as someone who thinks “outside the box” and people will tell you, you are different. You’ll know they mean it in a good way.’

R: ‘The point I am trying to make is that many autistic spectrum people usually have great genius in certain areas. In fact, the imbalance caused by the condition and the genius seem too often go together. It seems as if the imbalance in abilities may lead them to brilliance in others.’

### Limitations of study

I acknowledge that this study has limitations. Firstly, all the participants have had, or are receiving, the benefit of appropriate education and training, and live in urban areas. Thus, they may be considered to be the privileged few. Secondly, this study was undertaken out of personal interest to develop better strategies to inform my practice of work with people who have special needs. Therefore, it is a microview of (dis)ability within the workplace. Finally, it is important to note that whilst acknowledging that HIV and/or AIDS is a very important dimension of (dis)ability, especially in the South African context, I have set this aspect aside in an attempt to narrow the scope of this study. I believe that the impact of HIV and/or AIDS on success in the workplace is worthy of an independent study.

## Discussion

### Policy

Global awareness of disability is increasing. The United Nations Convention on the Rights of Persons with Disabilities (CRPD) specifically refers to the importance of international development in addressing the rights of people with disabilities and promotes their unrestricted integration in society. Despite the fact that South Africa has world class policies of good practice and has ratified the Convention on Rights of Persons with Disabilities (United Nations 2008; World Bank 2014), the plan of action to implement these policies is sometimes inadequate. The reality is that rights do not automatically enable people to live better lives.

One of the primary objectives of the Disability Policy Guideline (Department of Public Works 2010) was to encourage a tangible shift from policy to practice. The experiences described by the participants in this study illustrate that policy has not effectively influenced practice. Perhaps state institutions do not yet have the capacity and skills needed to action these policies (Dalton *et al*. 2012; Eide & Ingstad 2013; DHET 2014; DSD, DWCPD & UNICEF 2012; Van Deventer 2011). Hence, as members of society it is necessary for us all to work in our communities to raise awareness and provide appropriate support structures for people with (dis)abilities. Comprehensive psycho-educational prgrammes offered to all stakeholders would be of benefit in creating effective practice.

### Support structures

People do not exist in isolation; each individual is a member of a family and social community. There is abundant literature focussing on people with (dis)abilities and their families, how they interact with their environment and society, as well as their need for support (Buntinx 2013; Charlton 1998; Ingstad & Whyte 1995, 2007; Moore *et al*. 1998; Rocco 2011; Stone 2005; Watermeyer *et al*. 2006). The responses from the participants in this study confirm the need for support structures, and illustrate how people with (dis)abilities who receive support from family, friends and colleagues are enabled and thus become successful.

Despite the recommendation made in ‘Article 8 of CRPD’, which addresses Awareness Training, there is still evidence in the responses of a lack of appropriate training in society about disabilities and practical support. Buntinx (2013:13) defines support systems as ‘resources and strategies that aim to promote the development, education, interests and personal well-being of a person and enhance individual functioning’. The responses from the participants in this study illustrate that (dis)ability is a community endeavour, which requires a multidimensional and multidisciplinary approach. Awareness Training, using specific psycho-education programmes, could be provided for each person with a (dis)ability, as well as their families and prospective employers, who are their primary support system.

There is also evidence that the role of all professionals who work with people with (dis)abilities is useful when they identify unique strengths and develop individualised strategies to enhance the functioning of each person. Bach’s (2007) view that the role of the professional is not to determine if – but how people with (dis)abilities can live meaningfully and productively in a community. Buntinx (2013:15) suggest a four-phase approach which may be useful:

- assessment of individual strengths
- assessment of the person’s subjective expectations and objective needs
- linking personal goals to a range of related resources and action strategies
- evaluate support outcomes.

### Education and training

Taking into account ‘Article 24 of CRPD’ addressing education, and despite commitments from National Skills Development Strategy (NSDS) to increase opportunities for training and skills development for people with (dis)abilities South Africa is still far from achieving its goals in this regard (AfriNEAD 2009; DHET 2014; DWCPD 2013;WHO 2011). The DHET acknowledges the continued difficulty in providing sufficient capacity to accommodate and serve students with (dis)abilities, despite the fact that they have committed to making funding available. Clearly, more than funding is required to ameliorate the difficulties experienced by people with (dis)abilities in education and training.

Blade Nzimande continues to call for an adequate policy framework. He states that:

A strategic policy framework is necessary to guide the improvement of access to and success in post-school education and training (including in private institutions) for people with disabilities. The framework will create an enabling and empowering environment across the system. The framework will set norms and standards for the integration of students and staff with disabilities in all aspects of university or college life, including academic life, culture, sport and accommodation. (DHET 2014:8)

General psycho-education programmes would stimulate more knowledge about the needs of people with (dis)abilities and bring about a change in attitude.

### Change in attitude for the individual and society

Oliver (2009), amongst others, speaks about the ‘disablist’ attitude, which he describes as particularly disempowering. One of the six principles of Critical Disability Theory is that ‘Ableism is invisible’ (Rocco 2011:7). Therefore, it is imperative that people with (dis)abilities demonstrate their ‘ableism’, in order to be recognised. I wish to argue that people may not be able to demonstrate their ‘ableism’ if they struggle with self-esteem and are not recognised and encouraged to reach their potential.

All people entering the world of work benefit from having self-knowledge and being able to identify their natural talents, accept their limitations, and acquire market related skills (Marsay 2008). Several responses from participants in this study vividly illustrate how their (dis)ability can in fact be used as a strength. Indeed, many of the success stories told by these participants pivot on their ability to offer resistance to a ‘disabilist’ attitude. I wish to argue that many people who have (dis)abilities can be very competent members of the workforce if they are enabled to identify and develop their unique talent. Assisting people to establish positive self-regard, to see their intrinsic self-worth and to know their strengths and limitations is a priority. Wehmeyer and Little (2013:119) explain that people who are able to use accurate knowledge of themselves, value themselves and who know their strengths and weaknesses are able to capitalise on their knowledge.

Eide and Ingstad (2013) state that women with disabilities are worse off than men. Wehmeyer and Little (2013:125) describe findings of research studies which indicate that males show a higher degree of self-determination than females in certain cultures and societies. Could it be that in Africa, gender inequality may exacerbate the outcomes for people with (dis)abilities, especially women? Is there a link between a positive attitude of self, regard from others, and the ability to be self-determined?

### Self-determination

Eide and Ingstad (2013) discuss several issues which make it difficult for people with disabilities to live well. However, they note that many individuals with (dis)abilities still manage. I wish to argue, based on evidence discussed in literature and supported by the themes exposed in this study, that self-determination is crucial to the success of people with (dis)abilities.

According to Wehmeyer and Little (2013), self-determination actions are identified by four essential characteristics:

- a person acts autonomously
- behaviour is self-regulated
- the person initiates and responds to the event(s) in a psychologically empowered manner
- the person acts in a self-realising manner (p. 119).

The constructs of Positive Psychology include:

- quality of life
- self-determination
- adaptive behaviour
- optimism
- hope
- problem solving
- forgiveness
- gratitude
- spirituality.

Many of these constructs seem to be part of the fabric of the experiences told by the participants in this study, thereby highlighting these actions of self-determination. Wehmeyer and Little (2013) advocate that self-determnation can be learned. They suggest that further research around appropriate interventions to develop and investigate self determination would be useful in moving forward.

Self-determination is an essential part of success in the workplace for people who have (dis)abilities and is a product of both the person and the environment. According to Wehmeyer and Little (2013:121), ‘Self-determination is affected by environmental variables as well as by the knowledge, skills and beliefs expressed by the individual’. Hence, it is necessary to empower people who have (dis)abilities, with essential self-determination skills and assist them to seek out and create environments that offer the opportunity to actualise their potential using specific psycho-education programmes.

### Enabling environment

The responses from the participants in this study illustrate how people with (dis)abilities may not always be treated with sufficient knowledge, understanding or respect for their unique needs. Furthermore, it would appear that work environments continue to present accessibility difficulties. ‘Article 9 of CRPD’ addresses accessibility. Eide and Ingstad (2011:139) refer to ‘structural violence’ which includes not only buildings that are not easily accessible for those with disabilities, but also structures like the natural terrain that is inaccessible. These structures are not violent themselves, but become adversarial when nothing is done to overcome them as barriers. Rocco (2011:6) suggests that the environment becomes disabling when spaces are created without regard to the needs of people with (dis)abilities.

Therefore, once ubiquitous accessibility needs have been met, employers and employees need to communicate and collaborate to address specific (dis)abilities. It is necessary for employers to understand the specific needs of each individual, rather than making assumptions. In addition, it is necessary for people with (dis)abilities to be enabled to communicate their needs with confidence. Furthermore, it is essential for people with (dis)abilities to make their unique abilities visible to others.

It is interesting to note that whilst the majority of participants who applied for employment through a specialist agency were successful, some placements were unsuccessful. The participant who suffers from Rheumatoid Arthritis describes how she was required to work in a position she was physically unable to do. On the other hand, it is encouraging to note that another participant, who struggles with paraplegia, was able to qualify as a medical doctor due to understanding and accommodation of her condition. Thus, it may be useful for employers to adopt an all-encompassing biopsychosocial approach to disability, to pay keen attention to the ergonomics of the workplace and the surrounding area, as well as to make provision for special needs surrounding transport for people with (dis)abilities.

Clearly, there is a need for both general and specific psycho-education for both prospective employers, with regard to how they can accommodate the environment to suit the unique needs of a person with (dis)abilities, as well as the person with (dis)abilities himself or herself. The Supports Intensity Scale (Thompson *et al*. 2004) may be a useful tool to ascertain the specific needs of each person and can form the basis for an Individualised Support Plan (ISP) (Buntinx 2013).

## Conclusion

‘Article 27 of CRPD’ states that people are entitled to participate fully in the world of work. The stories of success described by the participants in this study highlight the following areas to be considered:

- Effective education and training is necessary to equip people who have (dis)abilities with appropriate marketable skills.
- Self-determination skills are essential to success and can be learned. Therefore, these skills need to be developed as part of specific psycho-educational intervention plans.
- Biopsychosocial support structures including attention to creating enabling work environments are essential for people with (dis)abilities to live, learn, work and play.
- It is necessary to find ways to co-construct an ‘ableist’ attitude in society. I wish to argue that more employment opportunities for people with (dis)abilities would stimulate hope for those who struggle with (dis)abilities and would work towards co-constructing an ‘ableist’ attitude in society.
- Both general and specific psycho-education programmes for employers, families and people with (dis)abilities that focus on how to address and support the needs of people with (dis)abilities is one of the most important approaches to consider that would ameliorate environmental and social obstacles.

It would appear, that despite good intentions and altruistic policies, there remains a lot that needs to be done to action good practice. This study describes the experiences of people who have education and live in urban areas. If they describe inadequate practice, then the question is posed, how much worse are conditions in rural areas, where people have limited access to education and health care services.

If we believe that the ‘cure’ to the problem of disability lies in exploring and making use of the strengths of the (dis)abled person as well as restructuring of society’s attitude towards (dis)ability, then, all people need to be trained according to their abilities, and it is the responsibility of the entire community to work towards providing enabling individualised support structures.
